# DNA Intercalating Near-Infrared Luminescent Lanthanide Complexes Containing Dipyrido[3,2-*a*:2′,3′-*c*]phenazine (dppz) Ligands: Synthesis, Crystal Structures, Stability, Luminescence Properties and CT-DNA Interaction

**DOI:** 10.3390/molecules25225309

**Published:** 2020-11-13

**Authors:** Aleksandar Savić, Anna M. Kaczmarek, Rik Van Deun, Kristof Van Hecke

**Affiliations:** 1 L^3^–Luminescent Lanthanide Lab, Department of Chemistry, Ghent University, Krijgslaan 281-S3, B-9000 Ghent, Belgium; aleksej95@yahoo.co.uk (A.S.); anna.kaczmarek@ugent.be (A.M.K.); 2 Chair of General and Inorganic Chemistry, Faculty of Chemistry, University of Belgrade, Studentski trg 12-16, 11000 Belgrade, Serbia; 3 NanoSensing Group, Department of Chemistry, Ghent University, Krijgslaan 281-S3, B-9000 Ghent, Belgium; 4 XStruct, Department of Chemistry, Ghent University, Krijgslaan 281-S3, B-9000 Ghent, Belgium

**Keywords:** lanthanide complexes, dipyrido[3,2-*a*:2′,3′-*c*]phenazine, crystal structures, NIR luminescence

## Abstract

In order to create near-infrared (NIR) luminescent lanthanide complexes suitable for DNA-interaction, novel lanthanide dppz complexes with general formula [Ln(NO_3_)_3_(dppz)_2_] (Ln = Nd^3+^, Er^3+^ and Yb^3+^; dppz = dipyrido[3,2-*a*:2′,3′-*c*]phenazine) were synthesized, characterized and their luminescence properties were investigated. In addition, analogous compounds with other lanthanide ions (Ln = Ce^3+^, Pr^3+^, Sm^3+^, Eu^3+^, Tb^3+^, Dy^3+^, Ho^3+^, Tm^3+^, Lu^3+^) were prepared. All complexes were characterized by IR spectroscopy and elemental analysis. Single-crystal X-ray diffraction analysis of the complexes (Ln = La^3+^, Ce^3+^, Pr^3+^, Nd^3+^, Eu^3+^, Er^3+^, Yb^3+^, Lu^3+^) showed that the lanthanide’s first coordination sphere can be described as a bicapped dodecahedron, made up of two bidentate dppz ligands and three bidentate-coordinating nitrate anions. Efficient energy transfer was observed from the dppz ligand to the lanthanide ion (Nd^3+^, Er^3+^ and Yb^3+^), while relatively high luminescence lifetimes were detected for these complexes. In their excitation spectra, the maximum of the strong broad band is located at around 385 nm and this wavelength was further used for excitation of the chosen complexes. In their emission spectra, the following characteristic NIR emission peaks were observed: for a) Nd^3+^: ^4^F_3/2_ → ^4^I_9/2_ (870.8 nm), ^4^F_3/2_ → ^4^I_11/2_ (1052.7 nm) and ^4^F_3/2_ → ^4^I_13/2_ (1334.5 nm); b) Er^3+^: ^4^I_13/2_ → ^4^I_15/2_ (1529.0 nm) c) Yb^3+^: ^2^F_5/2_ → ^2^F_7/2_ (977.6 nm). While its low triplet energy level is ideally suited for efficient sensitization of Nd^3+^ and Er^3+^, the dppz ligand is considered not favorable as a sensitizer for most of the visible emitting lanthanide ions, due to its low-lying triplet level, which is too low for the accepting levels of most visible emitting lanthanides. Furthermore, the DNA intercalation ability of the [Nd(NO_3_)_3_(dppz)_2_] complex with calf thymus DNA (CT-DNA) was confirmed using fluorescence spectroscopy.

## 1. Introduction

Lanthanide chemistry is a field of inorganic chemistry that is constantly evolving, particularly in recent decades, as a growing number of synthesized compounds are widely used in different areas. One of the most fascinating aspects of trivalent lanthanides is their unique luminescence properties [1,2,3]. They show characteristic narrow line-like emission peaks, ranging from the UV-visible (UV/Vis) to near-infrared (NIR), and in suitable hosts even in the mid-infrared (MIR) region, exhibiting relatively long luminescence lifetimes. Yet, a downside of trivalent lanthanides is that their *f*-*f* transitions are parity forbidden, and therefore their absorption coefficients are very low. This results in low emission intensities unless the compound is excited with high power sources, e.g., lasers. The design of lanthanide complexes with organic ligands, which are strongly absorbing chromophores, is an ideal way to overcome this drawback.

The unique properties (structural, optical and magnetic) of the lanthanide complexes allow them to be used in a wide range of applications [4]. In the field of biology and medicine these complexes are particularly investigated [5]. They are already being employed or show great potential as MRI contrast agents (Gd^3+^ compounds), fluorescent probes and bioresponsive cellular imaging agents, as well as artificial nucleases for cleavage of RNA or DNA [6,7,8]. Recently, studies have shown that other lanthanide compounds, such as oxide nanoparticles (e.g., CeO_2_), nanodrums and nanocrystals, as well as radioisotopes (e.g., ^177^Lu), represent very promising agents in cancer imaging and therapy [9,10]. Lanthanides usually have the +3 oxidation state and the significant redox stability of this oxidation state is very important for cellular applications, especially for hypoxic cancer cells [5]. The ability of rapid ligand exchange rates and strong Lewis acidity of the trivalent lanthanide ions and their complexes make them suitable in application as artificial nucleases for cleavage of RNA or DNA.

This study originated from our interest to design lanthanide complexes, which are highly emissive in the NIR region (780–2500 nm wavelength). In the aspect of biological applications, luminescent materials emitting in the NIR region of the electromagnetic spectrum have several advantages over visible emitting materials. Among the most important is that they are safer for biological tissues and result in better optical imaging resolution [11]. Biological tissue is also more transparent for NIR light than for visible or UV light, hence allowing imaging of deeper-lying tumor cells. Several of the trivalent lanthanide ions show NIR emission, given the right circumstances, such as Er^3+^, Ho^3+^, Tm^3+^, Pr^3+^, Yb^3+^, Nd^3+^, Sm^3+^ and Dy^3+^ [12]. Among them, Nd^3+^, Er^3+^ and Yb^3+^ are most often investigated.

Many efforts have already been invested in the design of relatively stable complexes of *f-*elements, which can be photoactivated upon light irradiation [13,14,15,16]. However, the design of highly emissive lanthanide complexes in the NIR region can be quite challenging and the choice of an appropriate organic ligand is crucial. We have focused our work on the design of lanthanide complexes with dipyrido[3,2-*a*:2′,3′-*c*]phenazine (dppz) as its triplet level is ideally suited for efficient sensitization of Nd^3+^, Er^3+^ and Yb^3+^ ions [17]. For example, a highly luminescent [Er(bta)_3_(dppz)] (Hbta = benzoyltrifluoroacetone) complex has previously been reported [18]. It exhibited stronger NIR emission than similar complexes prepared with the Hbta ligand and bipyridine, phenanthroline or dipyrido[3,2-*d*:2′,3′-f]quinoxaline. Recently, an [Yb(L)_3_(dppz)] (L = 3,5-heptanedione) complex has been reported, showing strong NIR luminescence and the incorporation of such complexes in poly(methyl methacrylate) (PMMA) thin films was studied for potential biological applications [19]. Ytterbium(III) complexes, such as [Yb(dppz)(DMF)_2_Cl_3_] and [Yb(dppz)(ttfa)_3_] (Httfa = 4,4,4-trifluoro-1-(2-thienyl)-1,3-butanedione) have been designed as NIR bioimaging probes using cooperative upconversion luminescence for theranostic application in the biological phototherapeutic window [20]. Another feature of polypyridyl ligands such as dppz is their ability to interact with DNA tracts [21], rendering them perfect candidates as NIR luminescent probes for cellular imaging and diagnostics, when combined with specific lanthanides. Here, in our pursuit to obtain compounds with strong NIR luminescence properties we have designed complexes, which consist of one lanthanide ion (Ln = Nd^3+^, Er^3+^ and Yb^3+^) and two dppz ligands. In all these complexes the lanthanide ions are ten-coordinated by two bidentate *N*,*N*-donor dppz ligands and three bidentate nitrate ions. We also synthesized analogous complexes with other lanthanide ions (Ln = Ce^3+^, Pr^3+^, Nd^3+^, Sm^3+^, Eu^3+^, Tb^3+^, Dy^3+^, Ho^3+^, Tm^3+^, Lu^3+^) and single crystals suitable for X-ray diffraction analysis were obtained for several of the Ln^3+^ complexes (Ln = La^3+^, Ce^3+^, Pr^3+^, Nd^3+^, Eu^3+^, Er^3+^,Yb^3+^, Lu^3+^). This shows that our synthetic approach, which is novel compared to other syntheses reported for phenazine complexes, is highly successful in yielding single crystals suitable for measurements of analogous structures. A detailed luminescence study of the [Nd(NO_3_)_3_(dppz)_2_], [Er(NO_3_)_3_(dppz)_2_] and [Yb(NO_3_)_3_(dppz)_2_] complexes was carried out and is presented in this paper. Additionally, the stability of the compounds in solution is verified by UV-Vis spectroscopy. Last, the intercalation ability of the [Nd(NO_3_)_3_(dppz)_2_] with calf thymus DNA (CT-DNA) was tested using fluorescence spectroscopy.

## 2. Results and Discussion

### 2.1. Synthesis of the Ligand and Complexes

The synthesis of the ligand precursor 1,10-phenanthroline-5,6-dione and of the dipyrido[3,2-*a*:2′,3′-*c*]phenazine ligand are presented in the supporting information (Schemes S1 and S2).

**Scheme 1:**
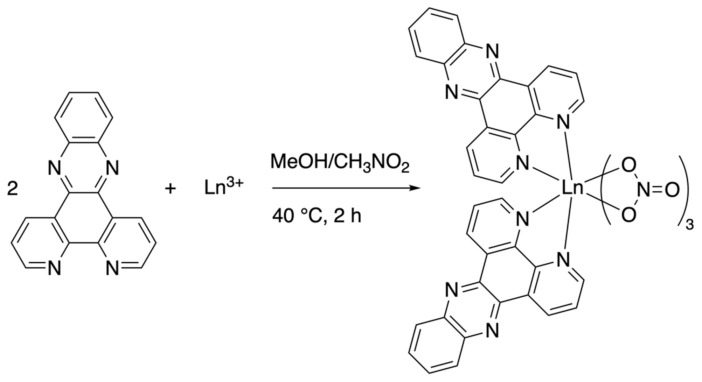
Synthesis of the [Ln(NO_3_)_3_(dppz)_2_] complexes (Ln = Ce^3+^, Pr^3+^, Nd^3+^, Sm^3+^, Eu^3+^, Tb^3+^, Dy^3+^, Ho^3+^, Er^3+^, Tm^3+^, Yb^3+^, Lu^3+^).

Complexes of general formula [Ln(NO_3_)_3_(dppz)_2_] (Ln = Ce^3+^, Pr^3+^, Nd^3+^, Sm^3+^, Eu^3+^, Tb^3+^, Dy^3+^, Ho^3+^, Er^3+^, Tm^3+^, Yb^3+^, Lu^3+^) were prepared by the reaction of the lanthanide nitrite salt and the dppz ligand in 1:2 molar ratio (Scheme 1). Reactions were performed in a mixture methanol/nitromethane at 40 °C, for 2 h. The complexes precipitated directly from the reaction mixture in moderate yields. The complexes are all soluble in dimethyl sulfoxide (DMSO) and dimethylformamide (DMF). The complexes [Ln(NO_3_)_3_(dppz)_2_] (Ln = La^3+^ and Gd^3+^) have previously been reported and hence are not included in the series [22].

### 2.2. Spectroscopic Study

1,10-Phenantroline-5,6-dione and the dipyrido[3,2-*a*:2′,3′-*c*]phenazine (dppz) ligand were characterized by IR and ^1^H-NMR spectroscopy. These compounds have already been described in literature and the positions of the bands in the IR spectra and chemical shift values in the ^1^H-NMR spectra are in accordance with the literature data [23,24] (see Materials and Methods, parts: 3.1., 3.3.1. and 3.3.2.). The IR spectrum of 1,10-phenantroline-5,6-dione showed characteristic bands at the following wavenumber positions: around 3062 cm^−1^ (C-H stretching vibrations), 1686 (C=O stretching vibrations), 1580–1562 and 1460–1418 cm^−1^ (C-C and C-N stretching vibrations in the ring). Characteristic bands of the dppz molecule were detected also at expected positions: around 3026 cm^−1^ (C-H stretching vibrations), 1589–1575 and 1485–1361 cm^−1^ (C-C and C-N stretching vibrations in the ring).

The IR spectra of all the complexes showed characteristic bands at the following wavenumber positions: around 3095 cm^−1^ (C-H stretching vibrations), 1600–1585 and 1500–1400 cm^−1^ (C-C and C-N stretching vibrations in the ring), 900–675 cm^−1^ (out-of-plane bends).

### 2.3. Single Crystal X-ray Analysis

A whole series of eight [Ln(NO_3_)_3_(dppz)_2_] complexes (Ln = La^3+^, Ce^3+^, Pr^3+^, Nd^3+^, Eu^3+^, Er^3+^, Yb^3+^ and Lu^3+^) were structurally characterized by single-crystal X-ray analysis. Although the [La(NO_3_)_3_(dppz)_2_] complex has previously been reported [22], its crystal structure has never been reported and is therefore included in the reported series. All structures were found to be isomorphic and exist as neutral, mononuclear species. Therefore, only the [Nd(NO_3_)_3_(dppz)_2_] complex is described as an example.

**Figure 1:**
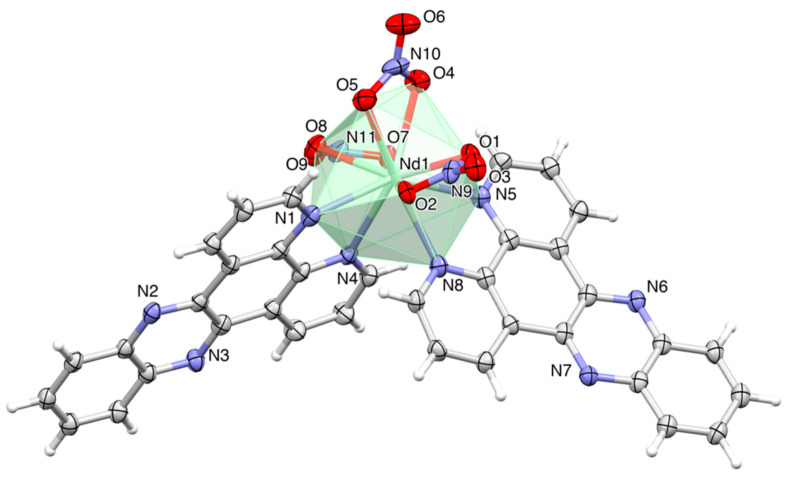
Asymmetric unit of the crystal structure of [Nd(NO_3_)_3_(dppz)_2_], showing thermal displacement ellipsoids at the 50% probability level, atom labeling scheme of the hetero atoms and polyhedron representation of the coordination environment of the Nd^3+^ ion.

The complex [Nd(NO_3_)_3_(dppz)_2_] crystallized in the centro-symmetric space group *P*2_1_/c, with one complete [Nd(NO_3_)_3_(dppz)_2_] complex as the asymmetric unit. The Nd^3+^ center is ten-coordinated by two bidentately coordinating *N,N’*-donor dppz ligands and three *O,O’*-bidentate nitrate ligands (Figure 1).

The coordination environment around Nd^3+^ can be best described as a bicapped dodecahedron geometry. As a consequence, the two dppz ligands are oppositely oriented, with an angle of 78.5° between the ligands. The Nd-O distances are in the range of 2.516(3) to 2.600(3) Å. The Nd-N distances are in the range of 2.590(3) to 2.629(3) Å.

In the packing, multiple π∙∙∙π stacking interactions are observed between the dppz aromatic rings (centroid-centroid distances of 3.653(2)–3.842(2)Å for distances <4Å). Furthermore, the nitrate ligands form N-O∙∙∙π interactions with several dppz aromatic rings (N-O∙∙∙centroid distances between 3.059(3)–3.931(3)Å).

An isostructural compound of the Eu^3+^ complex has previously been reported, however, this latter compound crystallized in the different space group *C*2/c, and featured inclusions of an extra dppz ligand and diethylether molecule in the crystal packing [25].

### 2.4. Photoluminescence Study

**Scheme 2:**
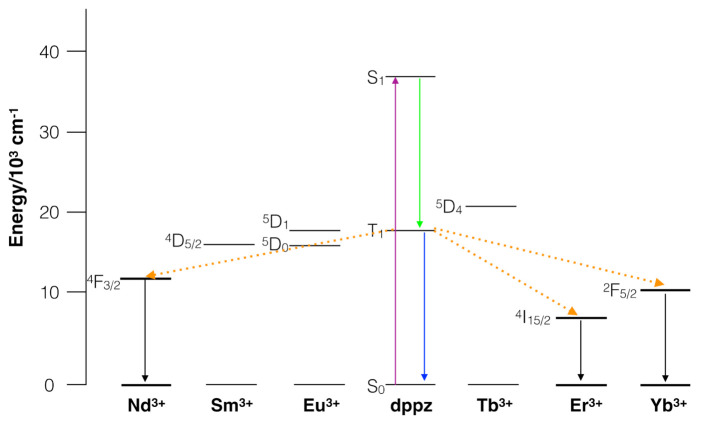
Schematic simplified energy level diagram of the dppz ligand and selected lanthanides employed in the study. Favorable energy transfer processes from the dppz to Ln^3+^ ions are shown as orange dotted arrows.

The [Ln(NO_3_)_3_(dppz)_2_] complexes containing spectroscopically active lanthanide ions were tested for their luminescence properties. All measurements were carried out in the solid state. It was observed that only the [Nd(NO_3_)_3_(dppz)_2_], [Er(NO_3_)_3_(dppz)_2_], [Yb(NO_3_)_3_(dppz)_2_] and [Eu(NO_3_)_3_(dppz)_2_] complexes displayed luminescence properties when excited into the maximum of the broad band originating from the dppz ligand. The NIR luminescence properties of the Nd^3+^, Er^3+^ and Yb^3+^ complexes are discussed in detail in this paper. For the sake of completeness, the excitation and emission spectra of the [Eu(NO_3_)_3_(dppz)_2_] complex are presented in the SI (Figures S1 and S2). The singlet and triplet energy levels of the dppz ligand have been previously reported in the literature (37000 and 18600 cm^−1^) [22]. It is known that the intersystem crossing process becomes effective when Δ*E* (^1^ππ*−^3^ππ*), the energy difference between the first excited singlet and triplet levels of the ligand, is more than 5000 cm^−1^ [26]. For the dppz ligand, the energy gap between the ^1^ππ* and ^3^ππ* amounts to 18400 cm^−1^, which indicates an effective intersystem crossing process. A study of the ligand’s triplet energy level and the resonant energy levels of the various lanthanides allows to predict whether efficient ligand-to-metal energy transfer can occur between the chosen ligand and a particular lanthanide ion. The ligand triplet energy level must be obviously higher than the resonant energy level of the lanthanide to result in adequate ligand to metal energy transfer. Of course, most likely the energy is not transferred directly to the emitting level of the lanthanide, but to higher levels, which then relax to the emitting levels via a nonradiative way. In Scheme 2 we present the singlet and triplet energy levels of dppz and of some selected spectroscopically active lanthanide ions. This clearly shows that the dppz ligand is very well suited for the design of NIR emitting Nd^3+^, Er^3+^ and Yb^3+^ complexes. On the other hand, the low triplet energy level of dppz makes it rather unfavorable as a sensitizer for most of the visible emitting lanthanide ions (e.g., Sm^3+^, Tb^3+^, Dy^3+^). Nevertheless, we were able to observe visible red emission for the [Eu(NO_3_)_3_(dppz)_2_] complex (studied in solid state, DMF and a DMF-H_2_O mixture; Figures S1 and S2).

**Figure 2:**
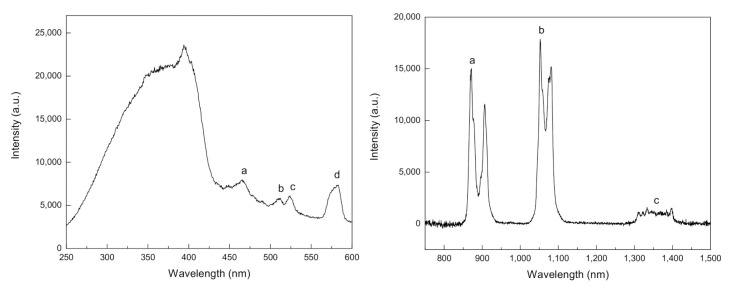
Excitation (**left**) and emission (**right**) steady-state spectra of the [Nd(NO_3_)_3_(dppz)_2_] complex measured at room temperature. The labeled peaks a–d and a–c, in the excitation and emission spectrum, respectively, have been assigned to appropriate transitions in Table 1.

**Figure 3:**
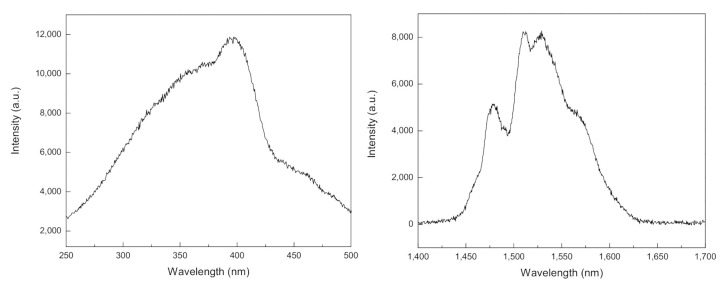
Excitation (**left**) and emission (**right**) steady-state spectra of the [Er(NO_3_)_3_(dppz)_2_] complex measured at room temperature. The broad emission band is referred to as peak “a” in Table 1.

**Figure 4:**
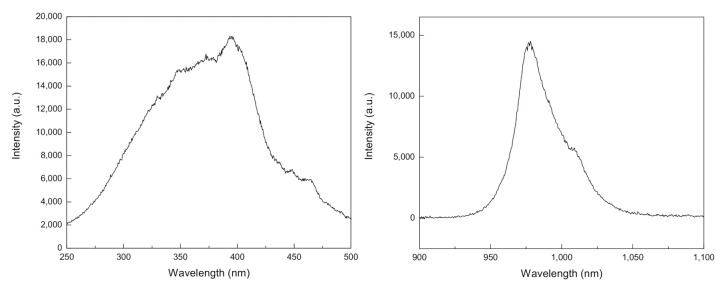
Excitation (**left**) and emission (**right**) steady-state spectra of the [Yb(NO_3_)_3_(dppz)2] complex measured at room temperature. The broad emission band is referred to as peak “a” in Table 1.

**Figure 5:**
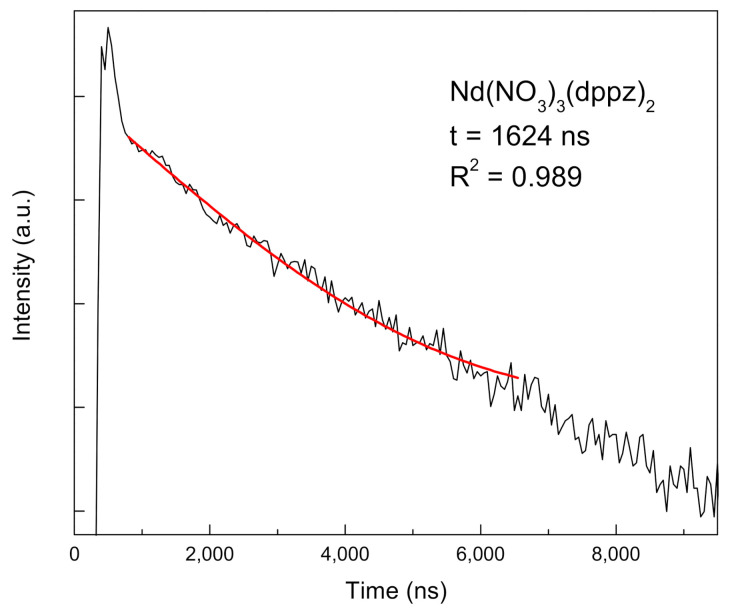
Decay profile of the [Nd(NO_3_)_3_(dppz)_2_] complex.

Steady-state excitation and emission spectra of the Nd^3+^, Er^3+^ and Yb^3+^ complexes were recorded at room temperature. For all three complexes, the excitation spectrum consisted of a strong broad band in the region 250–500 nm with a maximum at around 385.0 nm (Figure 2, Figure 3 and Figure 4). For [Nd(NO_3_)_3_(dppz)_2_], additionally several narrow sharp peaks were present, which could be assigned to the *f-f* transitions of Nd^3+^ (Table 1). When exciting the [Nd(NO_3_)_3_(dppz)_2_] complex at 385.0 nm the three characteristic NIR emission peaks of Nd^3+^ were recorded: ^4^F_3/2_ → ^4^I_9/2_ (870.8 nm), ^4^F_3/2_ → ^4^I_11/2_ (1052.7 nm), and ^4^F_3/2_ → ^4^I_13/2_ (1334.5 nm). The luminescence lifetime of the complex was also recorded. The decay curve of this compound as well as the [Er(NO_3_)_3_(dppz)_2_] and [Yb(NO_3_)_3_(dppz)_2_] complex could be well fitted using the single exponential equation:

(1) $$I=I_{0}\exp\left(-\frac{t}{\tau }\right)$$

where *I* and *I*_0_ are the luminescence intensities at time *t* and 0, respectively, and *τ* is the luminescence lifetime. The decay time of the [Nd(NO_3_)_3_(dppz)_2_] complex was determined to be 1624 ns or 1.62 µs (Figure 5).

**Figure 6:**
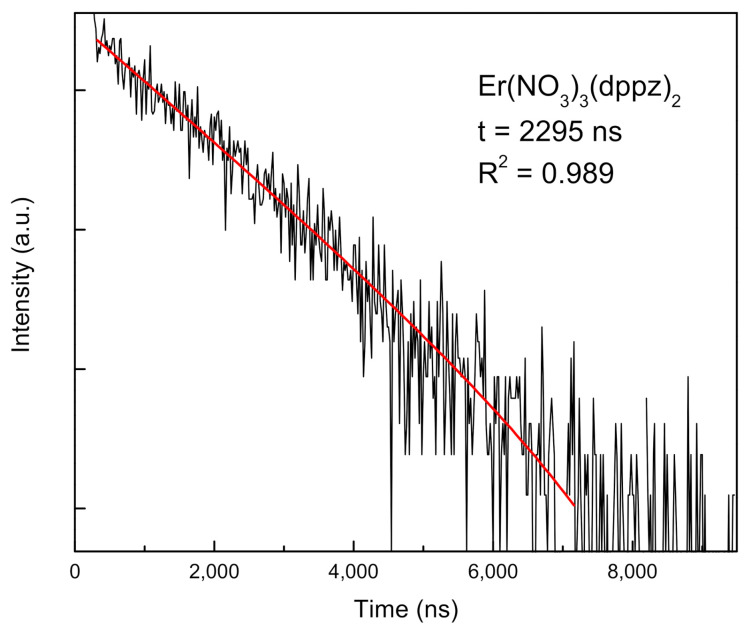
Decay profile of the Er(dppz)_2_(NO_3_)_3_ complex.

When exciting the [Er(NO_3_)_3_(dppz)_2_] complex at 385.0 nm one strong, broad peak located at 1529.0 nm, which can be assigned to the ^4^I_13/2_ →^4^I_15/2_ “telecom” transition, was observed. The luminescence lifetime was recorded for this complex and was determined as 2295 ns or 2.30 µs (Figure 6).

**Figure 7:**
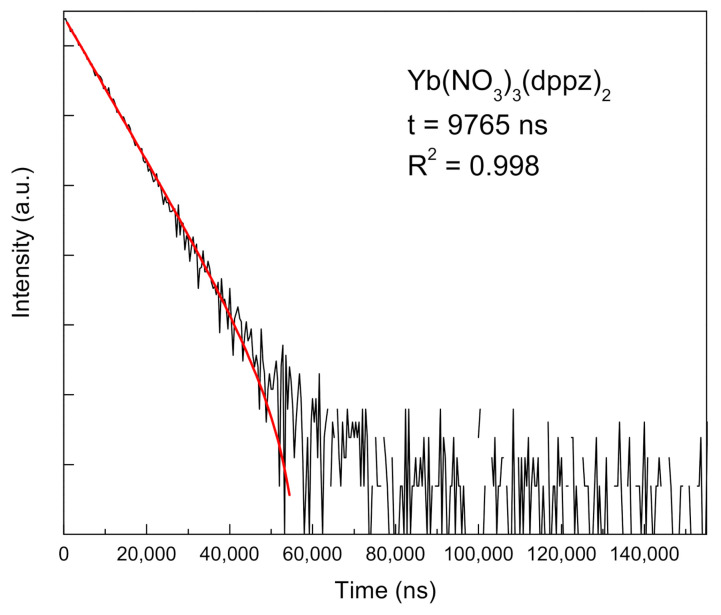
Decay profile of the [Yb(NO_3_)_3_(dppz)_2_] complex.

Exciting the [Yb(NO_3_)_3_(dppz)_2_] complex at 385.0 nm resulted in a strong broad peak with a maximum at 977.6 nm. This peak could be assigned to the ^2^F_5/2_ → ^2^F_7/2_ transition of Yb^3+^. The decay time of the complex was calculated to be 9765 nm or 9.77 µs (Figure 7).

The fact that the decay curves of the three complexes could be well fitted by monoexponential functions indicates the presence of single luminescence sites in the compounds, which is confirmed by the crystal structures.

The [Nd(NO_3_)_3_(dppz)_2_], [Er(NO_3_)_3_(dppz)_2_], [Yb(NO_3_)_3_(dppz)_2_] complexes show good luminescence properties, confirming that the dppz ligand is very well suited for the design of NIR emitting Nd^3+^, Er^3+^ and Yb^3+^ complexes. The dppz triplet level is well matched with the resonant energy levels of the two lanthanides Nd^3+^ and Er^3+^, allowing efficient energy transfer from the ligand to the lanthanide ions. Additionally, there are no water molecules in the first coordination sphere of the lanthanide minimizing non-radiative deactivation. To the best of our knowledge, so far only the luminescence properties of an [Er(bta)_3_(dppz)] complex have been reported for NIR-emitting lanthanide dppz complexes [18]. This compound has a higher decay time compared to our [Er(NO_3_)_3_(dppz)_2_] complex. This is quite expected due to the presence of a fluorinated Hbta ligand, as it is known that Er^3+^ complexes with fluorinated ligands have exceptionally long decay times (e.g., 16.8 µs for a complex with 1,1,1,3,5,5,5-heptafluoropentane-2,4-dione) [27,28].

### 2.5. UV/Vis Stability Tests

**Figure 8:**
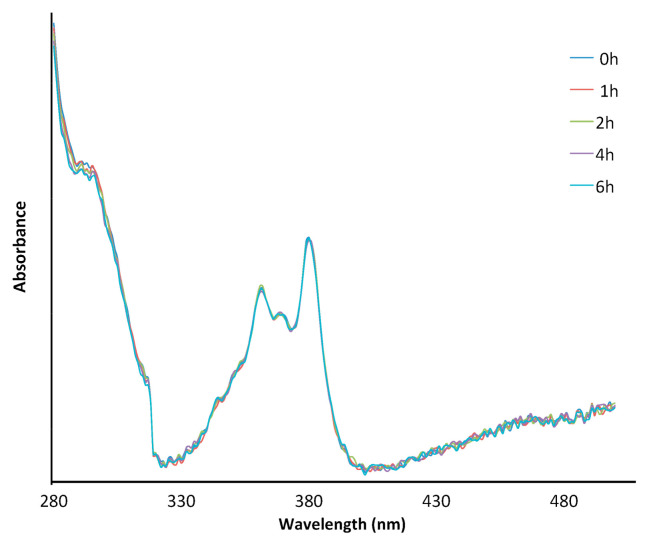
UV-Vis absorption spectra of the [Nd(NO_3_)_3_(dppz)_2_] complex carried out over a range of 6 h in DMF.

In order to enable the [Ln(NO_3_)_3_(dppz)_2_] complexes to have real application in biological environment it is necessary that they remain stable in solution over a certain period of time. Therefore, the stability of the [Nd(NO_3_)_3_(dppz)_2_], [Er(NO_3_)_3_(dppz)_2_] and [Yb(NO_3_)_3_(dppz)_2_] samples was tested over a period of 6 h. As can be found in literature the electronic absorption spectra of the dppz complexes in DMF show a ligand centered π→π* transition at around 265 nm. Two bands at 361 nm and 380 nm assigned to the n→π* transitions of the phenazine moiety are also present in the UV/Vis spectra [22,29]. In order to rule out decomposition of the complexes over time absorption spectral traces of the [Nd(NO_3_)_3_(dppz)_2_] in DMF were measured over a period of up to 6 h (Figure 8). No significant changes are visible in the spectra. Similar experiments were carried out for the [Er(NO_3_)_3_(dppz)_2_] and [Yb(NO_3_)_3_(dppz)_2_] compounds and exhibited no appreciable changes in the UV/Vis spectra over time (Figures S3 and S4). Our UV-Vis spectra are recorded in the 280–500 nm region to observe the n→π* transitions of the phenazine moiety. The complexes were also soluble in a mixture of DMF/Tris buffer (5 mM Tris-HCl, 5 mM NaCl, pH 7.2) and weakly soluble in only the Tris buffer (Figures S4 and S5).

To further investigate the stability of these [Ln(NO_3_)_3_(dppz)_2_] complexes, we have analyzed the [Eu(NO_3_)_3_(dppz)_2_] complex in different environments. It is known, that in europium compounds the splitting of the ^5^D_0_ → ^7^F_0–6_ peaks contains information on the europium ion’s environment [2]. Therefore, europium is often referred to as a structural probe. We have performed measurements of the [Eu(NO_3_)_3_(dppz)_2_] complex in solid, DMF and 50%DMF + 50%H_2_O mixture (see Figures S1 and S2). All emission spectra have been recorded upon exciting into the maximum of the dppz ligand’s absorption band at 370 nm. As can be observed, there are some changes in the emission spectra measured in the different environments, but it is clear that the red emission of the Eu^3+^ is retained even in 50%DMF + 50%H_2_O. However, the excitation spectra recorded in the three cases look remarkably similar (apart from the ^5^L_6_ ← ^7^F_0_ peak slightly below 400 nm in the solid state spectrum). This clearly indicates that the Eu^3+^ ion remains coordinated by at least one of the dppz ligands even after dissolution in DMF and after adding additional distilled water. In order to investigate whether indeed a different complex would be present after dissolution in DMF or DMF/water mixture, the luminescence decay traces were recorded in H_2_O and D_2_O to determine the number of directly coordinated water molecules using the Horrock’s equation [30,31] (Figures S7–S10). In fact, DMF/H_2_O 50/50 and DMF/D_2_O 50/50 mixtures were used as the complex isn’t sufficiently soluble in pure H_2_O or D_2_O. This yielded a number of coordinated water molecules of 3.73, pointing to the coordination of three or four water molecules to the Eu^3+^ ion. Given the fact that at least one dppz ligand should remain coordinated to the Eu^3+^ ion, simply replacing one dppz ligand with water molecules would not yield a number of three to four directly coordinated water molecules. As the excitation spectra are almost identical in the three cases, it is more likely that some of the nitrate ions that coordinate to the Eu^3+^ ion are replaced by water or DMF molecules (the nitrates remaining in the second coordination sphere for example) upon dissolution, resulting in a local coordination site at the Eu^3+^ ion that is less dissimilar to the original one in the solid state. It can therefore be assumed that indeed changes in the complex’s composition may occur upon dissolution in DMF or a DMF/water mixture, but most likely this does not involve drastic rearrangements such as the loss of the dppz ligands. As all reported complexes are isostructural, we expect similar behavior of the whole series of compounds.

### 2.6. CT-DNA Interaction Tests

In order to assess if the obtained [Ln(NO_3_)_3_(dppz)_2_] complexes show DNA interaction ability we carried out an ethidium bromide (EthB) displacement assay with CT-DNA in the presence of the [Nd(NO_3_)_3_(dppz)_2_] complex as an example. EthB is employed as a spectral probe, which shows enhanced emission intensity when intercalated to DNA and reduced emission intensity in the free state in water or buffer medium (due to solvent quenching of the fluorescence). The competitive interaction of the [Nd(NO_3_)_3_(dppz)_2_] complex, which could result in the displacement of the CT-DNA intercalated EthB, was monitored using fluorescence spectroscopy.

**Figure 9:**
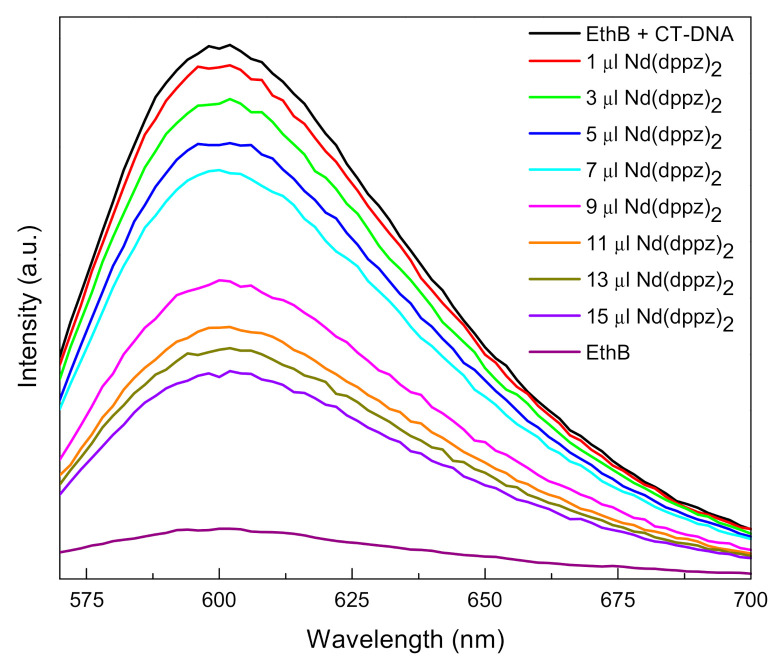
Emission spectral traces of EthB bound CT-DNA with increasing [Nd(NO_3_)_3_(dppz)_2_] additions (1–15 µL range; 14 mM). The measurements were carried out in 5 mM Tris-HCl/NaCl buffer (pH = 7.2) at RT; λ_ex_ = 546 nm, λ_em, max._ = 603 nm.

Figure 9 presents the emission spectra of EthB, EthB pretreated with CT-DNA, and EthB pretreated with CT-DNA and increasing additions of the [Nd(NO_3_)_3_(dppz)_2_] complex. As noticed, based on the figure, with increasing [Nd(NO_3_)_3_(dppz)_2_] concentration, the EthB emission intensity decreases. This means that the [Nd(NO_3_)_3_(dppz)_2_] complex shows sufficient interaction ability to the CT-DNA. With increasing [Nd(NO_3_)_3_(dppz)_2_] concentration an increased amount if EthB is displaced from the CT-DNA and is hence in the free state in the buffer. Due to solvent quenching, a drop in the emission intensity is clearly observed.

The apparent binding constant *K*_app_ of the [Nd(NO_3_)_3_(dppz)_2_] complex to the CT-DNA was determined using the equation *K*_EthB_ [EthB] = *K*_app_ *C*_50_, where *K*_EthB_ is the binding constant of EthB (1.0 10^7^ M^−1^, [EthB] is the EthB concentration (1.27 mM) and *C*_50_ is the concentration of the [Nd(NO_3_)_3_(dppz)_2_] complex at 50% reduction of the fluorescence intensity of the EthB (323.6 μM; based on the Stern–Volmer plot of I/I_0_ vs [complex]; see Supplementary Materials) [20,32]. The *K*_app_ equals 3.9 10^7^ M^−1^, indicating high affinity to CT-DNA, most probably through a DNA intercalative mode. A similar value of 1.2 10^7^ M^−1^ was found for a [Pt(pydppz)Cl]Cl (pydppz = 6-(2-pyridyl)-dipyrido-[3,2-*a*:2′,3′-*c*]phenazine) complex [33], while lower values of ∼1.3–6.0 10^6^ M^−1^ have often been reported for other lanthanide dppz complexes [20,25,29].

## 3. Materials and Methods

### 3.1. Synthesis

All chemicals (analytical grade) were purchased from Sigma Aldrich, VWR, Acros Organics or Merck Chemicals and used without further purification. The calf thymus DNA (CT-DNA) was Type 1, fibers, and was purchased from Sigma Aldrich. The precursor ligand, 1,10-phenantroline-5,6-dione and the dipyrido[3,2-*a*:2′,3′-*c*]phenazine ligand were synthesized according to literature procedures with slight modifications [23,24].

### 3.2. Characterization

Elemental analysis (C, H, N) was carried out with a Thermo Scientific Flash 2000 Series CHNS/O analyzer.

IR spectra were recorded from 4000 to 650 cm^−1^, on a Thermo Scientific FT-IR spectrometer (type Nicolet 6700), equipped with a DRIFTS-cell, using KBr as a non-absorbing matrix.

Photoluminescence measurements were recorded on an Edinburgh Instruments FLSP920 UV/Vis-NIR spectrofluorometer, using a 450 W xenon lamp as the steady state excitation source and a Hamamatsu R5509-72 photomultiplier operating at −80 °C. For the Eu^3+^ spectrum, a Hamamatsu R928P PMT was used, for the 200–900 nm range. The time-resolved measurements were performed using a Continuum Surelite I laser (450 mJ @1064 nm), operating at a repetition rate of 10 Hz and using the third harmonic (355 nm) as the excitation source, and the photomultiplier detector mentioned above. For all compounds, the luminescence of solid samples was recorded. Small amounts of the powder were placed between quartz plates.

UV/Vis measurements were performed on a Lambda 950 UV/Vis-NIR spectrophotometer from Perkin Elmer employing quartz cuvettes with a 10 mm path length.

NMR spectra were recorded in DMSO-d6, on a Bruker Avance 300 MHz or 500 MHz and all chemical shifts are given relative to tetramethylsilane (TMS).

### 3.3. Synthesis of the Ligand

#### 3.3.1. Synthesis of the Ligand Precursor 1,10-phenanthroline-5,6-dione (Phendione)

1,10-Phenanthroline (55.5 mmol) was added in small portions under stirring to sulfuric acid (65%, 80 mL) and left to dissolve at room temperature. Potassium bromate (62 mmol) was added in portions over a period of 3 h and the mixture was stirred at room temperature for 24 h. Then, the mixture was poured over ice and was neutralized to pH 7 using sodium carbonate. The mixture was extracted with chloroform and the organic phase was dried over magnesium sulfate during the night. The solution was then filtered and evaporated to dryness. The crude product was recrystallized from methanol to obtain the desired yellow product.

Yield: 8.4 g (71.8%).

IR (KBr, cm^−1^): 3062, 1686, 1580, 1562, 1460, 1418, 1290, 1205, 1116, 923, 816, 747.

^1^H-NMR (500 MHz, DMSO-*d*_6_) δ ppm 7.70 (dd, 2H, *J* = 8.1, 4.8 Hz); 8.40 (dd, 2H, *J* = 8.1, 1.8 Hz); 8.99 (dd, 2H, *J* = 4.8, 1.8 Hz).

#### 3.3.2. Synthesis of the Ligand Dipyrido[3,2-a:2′,3′-c]phenazine (dppz)

Phendione (0.24 mmol) was dissolved in ethanol (96%, 4 mL) at 75 °C, *o*-phenylenediamine (0.24 mmol) was added and the mixture was heated for 30 min under reflux. After cooling to room temperature, the dppz ligand was precipitated. The product was isolated, washed with methanol and dried under vacuum.

Yield: 50 mg (73.5%).

IR (KBr, cm^−1^): 3026, 1589, 1575, 1485, 1477, 1445, 1405, 1361, 1125, 1208, 1112, 1072, 1053, 1029, 922, 847, 836, 807, 739.

^1^H-NMR (500 MHz, DMSO-*d*_6_) δ ppm 7.97 (dd, 2H, *J* = 8.1, 4.4 Hz); 8.09 (m, 2H); 8.42 (m, 2H); 9.24 (dd, 2H, *J* = 4.3, 1.8 Hz); 9.56 (m, 2H).

### 3.4. Synthesis of the Complexes

The starting lanthanide nitrate salt (0.06 mmol) was dissolved in a mixture of nitromethane/methanol (1/1, 12 mL) and heated at 40 °C. To this, a solution of dppz ligand (0.12 mmol) in warm methanol (8 mL) was added dropwise with stirring, and the reaction mixture was left for 2 h, at 40 °C. During that period, the desired complex precipitated. The product was filtered off, washed with nitromethane (2 mL), water (1 mL) and ether (2 mL) and dried under reduced pressure. The filtrate was set up for crystallization via vapor diffusion of acetonitrile or isopropanol as antisolvents.

(C_18_H_10_N_4_)_2_Ce(NO_3_)_3_·H_2_O (**1**).

Yield: 30 mg (55%). Anal. Calcd. for (C_18_H_10_N_4_)_2_Ce(NO_3_)_3_·H_2_O, %: C 47.58, H 2.44, N 16.95. Found, %: C 47.79, H 2.00, N 16.35. IR (KBr, cm^−1^): 3100–3580, 3095, 1586, 1578, 1494, 1441, 1420, 1364, 1340, 1303, 1231, 1137, 1128, 1079, 1047, 1034, 844, 813, 795, 776.

(C_18_H_10_N_4_)_2_Pr(NO_3_)_3_·0.25H_2_O) (**2**).

Yield: 35 mg (65%). Anal. Calcd. for (C_18_H_10_N_4_)_2_Pr(NO_3_)_3_·0.25H_2_O, %: C 48.26, H 2.31, N 17.20. Found, %: C 47.94, H 2.04, N 16.65. IR (KBr, cm^−1^): 3570, 3095, 1599, 1586, 1577, 1498, 1440, 1420, 1364, 1340, 1303, 1231, 1137, 1128, 1079, 1046, 1032, 844, 822, 795, 762.

(C_18_H_10_N_4_)_2_Nd(NO_3_)_3_·0.5H_2_O (**3**).

Yield: 31 mg (57%). Anal. Calcd. for (C_18_H_10_N_4_)_2_Nd(NO_3_)_3_·0.5H_2_O, %: C 47.84, H 2.34, N 17.05. Found, %: C 47.67, H 2.00, N 16.45. IR (KBr, cm^−1^): 3094, 1597, 1584, 1576, 1493, 1442, 1421, 1365, 1340, 1302, 1236, 1137, 1129, 1079, 1048, 1033, 845, 822, 791, 761.

(C_18_H_10_N_4_)_2_Sm(NO_3_)_3_·0.75H_2_O (**4**).

Yield: 30 mg (55%). Anal. Calcd. for (C_18_H_10_N_4_)_2_Sm(NO_3_)_3_·0.75H_2_O, %: C 47.28, H 2.37, N 16.85. Found, %: C 46.96, H 1.89, N 16.29. IR (KBr, cm^−1^): 3100-3560, 3095, 1600, 1586, 1578, 1497, 1441, 1420, 1363, 1340, 1302, 1231, 1137, 1128, 1079, 1047, 1034, 843, 822, 795, 776, 762.

(C_18_H_10_N_4_)_2_Eu(NO_3_)_3_·0.5H_2_O (**5**).

Yield: 36 mg (66%). Anal. Calcd. for (C_18_H_10_N_4_)_2_Eu(NO_3_)_3_·0.5H_2_O, %: C 47.43, H 2.32, N 16.90. Found, %: C 47.28, H 1.88, N 16.34. IR (KBr, cm^−1^): 3400-3560, 3095, 1600, 1586, 1577, 1497, 1490, 1473, 1441, 1420, 1363, 1340, 1309, 1230, 1137, 1127, 1079, 1047, 1035, 844, 822, 795, 775, 762.

(C_18_H_10_N_4_)_2_Tb(NO_3_)_3_·0.25H_2_O (**6**).

Yield: 35 mg (64%). Anal. Calcd. for (C_18_H_10_N_4_)_2_Tb(NO_3_)_3_·0.25H_2_O, %: C 47.30, H 2.26, N 16.86. Found, %: C 46.89, H 1.97, N 16.34. IR (KBr, cm^−1^): 3400-3560, 3096, 1600, 1589, 1576, 1496, 1490, 1473, 1441, 1419, 1364, 1339, 1308, 1231, 1138, 1126, 1077, 1047, 1036, 844, 822, 795, 776, 762.

(C_18_H_10_N_4_)_2_Dy(NO_3_)_3_·0.25H_2_O (**7**).

Yield: 36 mg (65%). Anal. Calcd. for (C_18_H_10_N_4_)_2_Dy(NO_3_)_3_·0.25H_2_O, %: C 47.12, H 2.25, N 16.79. Found, %: C 46.60, H 1.96, N 16.34. IR (KBr, cm^−1^): 3450-3560, 3095, 1600, 1586, 1577, 1497, 1441, 1420, 1363, 1340, 1301, 1230, 1188, 1127, 1078, 1046, 1033, 822, 813, 776, 762, 737.

(C_18_H_10_N_4_)_2_Ho(NO_3_)_3_·0.25H_2_O (**8**).

Yield: 31 mg (56%). Anal. Calcd. for (C_18_H_10_N_4_)_2_Ho(NO_3_)_3_·0.25H_2_O, %: C 47.00, H 2.25, N 16.75. Found, %: C 46.41, H 1.96, N 16.20. IR (KBr, cm^−1^): 3100–3560, 3096, 1600, 1589, 1574, 1494, 1443, 1423, 1361, 1341, 1302, 1234, 1189, 1125, 1074, 1044, 1031, 821, 815, 771, 760, 734.

(C_18_H_10_N_4_)_2_Er(NO_3_)_3_·0.25H_2_O (**9**).

Yield: 37 mg (67%). Anal. Calcd. for (C_18_H_10_N_4_)_2_Er(NO_3_)_3_·0.25H_2_O, %: C 46.88, H 2.24, N 16.70. Found, %: C 46.49, H 1.98, N 16.14. IR (KBr, cm^−1^): 3400-3560, 3097, 1601, 1586, 1577, 1498, 1489, 1443, 1420, 1363, 1339, 1312, 1231, 1189, 1138, 1080, 1047, 1029, 822, 812, 773, 763, 737.

(C_18_H_10_N_4_)_2_Tm(NO_3_)_3_·0.5H_2_O (**10**).

Yield: 34 mg (61%). Anal. Calcd. for (C_18_H_10_N_4_)_2_Tm(NO_3_)_3_·0.5H_2_O, %: C 46.57, H 2.28, N 16.59. Found, %: C 46.30, H 1.88, N 16.02. IR (KBr, cm^−1^): 3550, 3096, 1601, 1586, 1578, 1498, 1489, 1441, 1420, 1364, 1340, 1309, 1231, 1188, 1138, 1127, 1079, 1047, 1035, 1026, 822, 813, 777, 762, 738.

(C_18_H_10_N_4_)_2_Yb(NO_3_)_3_·0.5H_2_O (**11**).

Yield: 29 mg (52%). Anal. Calcd. for (C_18_H_10_N_4_)_2_Yb(NO_3_)_3_·0.5H_2_O, %: C 46.36, H 2.27, N 16.52. Found, %: C 46.10, H 1.99, N 15.99. IR (KBr, cm^−1^): 3350-3560, 3093, 1600, 1584, 1579, 1495, 1488, 1440, 1421, 1364, 1341, 1313, 1230, 1186, 1132, 1126, 1073, 1046, 1036, 1025, 821, 815, 773, 761, 738.

(C_18_H_10_N_4_)_2_Lu(NO_3_)_3_·0.5H_2_O (**12**).

Yield: 28 mg (50%). Anal. Calcd. for (C_18_H_10_N_4_)_2_Lu(NO_3_)_3_·0.5H_2_O, %: C 46.26, H 2.26, N 16.49. Found, %: C 46.09, H 1.96, N 15.97. IR (KBr, cm^−1^): 3350-3550, 3095, 1600, 1582, 1578, 1492, 1487, 1441, 1425, 1362, 1342, 1314, 1231, 1184, 1131, 1123, 1071, 1046, 1036, 1024, 821, 813, 777, 767, 732.

^1^H-NMR (300 MHz, DMSO-*d*_6_) δ ppm 7.95 (dd, 2H, *J* = 8.3, 4.5 Hz); 8.06 (m, 2H); 8.38 (m, 2H); 9.22 (dd, 2H, *J* = 4.5, 1.9 Hz); 9.54 (dd, 2H, *J* = 8.3, 1.9 Hz).

^13^C-NMR (75 MHz, DMSO-*d*_6_) δ 124.6; 127.0; 129.2; 131.3; 133.1; 140.8; 141.8; 147.8; 152.4.

### 3.5. Solubility and Stability of Complexes

The [Ln(NO_3_)_3_(dppz)_2_] complexes were soluble in DMF and DMSO. The solution stability of the [Ln(NO_3_)_3_(dppz)_2_] (Ln = Nd^3+^, Er^3+^,Yb^3+^) in DMF was tested. The solubility in DMF/buffer mixtures (5 mM Tris-HCl, 5 mM NaCl, pH 7.2) was additionally tested.

### 3.6. Single Crystal X-ray Diffraction Analysis

For all reported structures, X-ray intensity data were collected, at 100 K, on a Rigaku Oxford Diffraction Supernova Dual Source (Cu at zero) diffractometer equipped with an Atlas charge-coupled device (CCD) detector using ω scans and CuKα (λ = 1.54184 Å) radiation. The images were interpreted and integrated with the program CrysAlisPro (Rigaku Oxford Diffraction) [34]. Using Olex2 [35], the structures were solved by direct methods using the ShelXS structure solution program and refined by full-matrix least-squares on F^2^ using the ShelXL program package [36,37]. Nonhydrogen atoms were anisotropically refined and the hydrogen atoms in the riding mode and isotropic temperature factors fixed at 1.2 times U(eq) of the parent atoms. For all reported structures, a correction for diffuse effects, due to the inclusion of disordered water molecules, was made using the SQUEEZE routine in PLATON [38].

Crystal data for compound: C_36_H_20_LaN_11_O_9_, M = 889.54, monoclinic, space group P2_1_/c (No. 14), a = 9.5038(4) Å, b = 14.3572(6) Å, c = 25.1669(12) Å, β = 91.800(4)°, V = 3432.3(3) Å^3^, Z = 4, T = 100 K, ρ_calc_ = 1.721 g cm^−3^, μ(Cu-Kα) = 10.278 mm^−1^, F(000) = 1768, 29511 reflections measured, 6908 unique (R_int_ = 0.0951) which were used in all calculations. The final R1 was 0.0666 (I>2σ(I)) and wR2 was 0.2016 (all data).

Crystal data for compound (**1**): C_36_H_20_CeN_11_O_9_, M = 890.75, monoclinic, space group P2_1_/c (No. 14), a = 9.4873(3) Å, b = 14.4189(5) Å, c = 25.1531(6) Å, β = 91.896(2)°, V = 3438.97(18) Å^3^, Z = 4, T = 100 K, ρ_calc_ = 1.720 g cm^−3^, μ(Cu-Kα) = 10.886 mm^−1^, F(000) = 1772, 20269 reflections measured, 6902 unique (R_int_ = 0.0674) which were used in all calculations. The final R1 was 0.0518 (I>2σ(I)) and wR2 was 0.1398 (all data).

Crystal data for compound (**2**): C_36_H_20_N_11_O_9_Pr, M = 891.54, monoclinic, space group P2_1_/c (No. 14), a = 9.46171(12) Å, b = 14.4654(2) Å, c = 25.0861(3) Å, β = 91.9166(12)°, V = 3431.55(8) Å^3^, Z = 4, T = 100 K, ρ_calc_ = 1.726 g cm^−3^, μ(Cu-Kα) = 11.550 mm^−1^, F(000) = 1776, 26255 reflections measured, 6881 unique (R_int_ = 0.0497) which were used in all calculations. The final R1 was 0.0352 (I>2σ(I)) and wR2 was 0.0924 (all data).

Crystal data for compound (**3**): C_36_H_20_N_11_NdO_9_, M = 894.87, monoclinic, space group P2_1_/c (No. 14), a = 9.4493(2) Å, b = 14.5407(4) Å, c = 24.9067(7) Å, β = 92.053(3)°, V = 3419.97(15) Å^3^, Z = 4, T = 100 K, ρ_calc_ = 1.738 g cm^−3^, μ(Cu-Kα) = 12.244 mm^−1^, F(000) = 1780, 23245 reflections measured, 6884 unique (R_int_ = 0.0409) which were used in all calculations. The final R1 was 0.0374 (I>2σ(I)) and wR2 was 0.1018 (all data).

Crystal data for compound (**5**): C_36_H_20_EuN_11_O_9_, M = 902.60, monoclinic, space group P2_1_/c (No. 14), a = 9.3990(2) Å, b = 14.6262(4) Å, c = 24.8352(6) Å, β = 91.972(2)°, V = 3412.11(14) Å^3^, Z = 4, T = 100 K, ρ_calc_ = 1.757 g cm^−3^, μ(Cu-Kα) = 13.820 mm^−1^, F(000) = 1792, 21978 reflections measured, 6860 unique (R_int_ = 0.0667) which were used in all calculations. The final R1 was 0.0480 (I>2σ(I)) and wR2 was 0.1326 (all data).

Crystal data for compound (**9**): C_36_H_20_ErN_11_O_9_, M = 917.89, monoclinic, space group P2_1_/c (No. 14), a = 9.3230(2) Å, b = 14.7818(7) Å, c = 24.6058(10) Å, β = 91.848(3)°, V = 3389.2(2) Å^3^, Z = 4, T = 100 K, ρ_calc_ = 1.799 g cm^−3^, μ(Cu-Kα) = 5.262 mm^−1^, F(000) = 1812, 19595 reflections measured, 6746 unique (R_int_ = 0.0773) which were used in all calculations. The final R1 was 0.0597 (I>2σ(I)) and wR2 was 0.1667 (all data).

Crystal data for compound (**11**): C_36_H_20_N_11_O_9_Yb, M = 923.67, monoclinic, space group P2_1_/c (No. 14), a = 9.3018(3) Å, b = 14.8195(5) Å, c = 24.5502(6) Å, β = 91.654(2)°, V = 3382.79(18) Å^3^, Z = 4, T = 100 K, ρ_calc_ = 1.814 g cm^−3^, μ(Cu-Kα) = 5.781 mm^−1^, F(000) = 1820, 20841 reflections measured, 6619 unique (R_int_ = 0.0766) which were used in all calculations. The final R1 was 0.0498 (I>2σ(I)) and wR2 was 0.1331 (all data).

Crystal data for compound (**12**): C_36_H_20_LuN_11_O_9_, M = 925.60, monoclinic, space group P2_1_/c (No. 14), a = 9.2924(2) Å, b = 14.8631(5) Å, c = 24.4840(7) Å, β = 91.488(2)°, V = 3380.44(17) Å^3^, Z = 4, T = 100 K, ρ_calc_ = 1.819 g cm^−3^, μ(Cu-Kα) = 6.258 mm^−1^, F(000) = 1824, 25239 reflections measured, 6807 unique (R_int_ = 0.0590) which were used in all calculations. The final R1 was 0.0375 (I>2σ(I)) and wR2 was 0.1012 (all data).

CCDC2034576-2034583 contain the supplementary crystallographic data for this paper and can be obtained free of charge via www.ccdc.cam.ac.uk/conts/retrieving.html (or from the Cambridge Crystallographic Data Centre, 12, Union Road, Cambridge CB2 1EZ, UK; fax: +44-1223-336033; or deposit@ccdc.cam.ac.uk).

### 3.7. CT-DNA Binding Study

CT-DNA was dissolved in Tris-HCl/NaCl buffer (5 mM, pH = 7.2). The DNA was left for several hours to completely dissolve, no sonication was used. The ratio of the absorbance values of the CT-DNA at 260 and 280 nm was ∼1.9, confirming the CT-DNA is free from protein impurities. The CT-DNA concentration was determined by absorption spectroscopy using a molar absorption coefficient value of 6600 M^−1^ cm^−1^ at 260 nm for CT-DNA [29]. The initial concentration of the CT-DNA was determined to 14.15 mM. The EthB was also dissolved in the Tris-HCl/NaCl buffer (5 mM, pH = 7.2) to obtain an initial concentration of 2.54 mM. Subsequently, 250 μL of both the CT-DNA and EthB solutions were added in a quartz cuvette (Starna cuvette type 23/Q/10) with a path length of 10 mm yielding a [CT-DNA] = 7.26 mM and [EthB] = 1.27 mM. The tested [Nd(NO_3_)_3_(dppz)_2_] complex was dissolved in DMF (14 mM) and the appropriate amount was used for the DNA binding study. The competitive binding assay from EthB displacement was carried out by measuring the emission intensities of EthB bound to CT-DNA solution with the gradual increase of the complex concentrations (27.9–406.9 μM; taking into account the dilution). The EthB was excited at 546 nm and observed at 603 nm (emission maximum). EthB showed very weak emission in the Tris-buffer due to fluorescent quenching of the free EthB in buffer. CT-DNA-bound EthB showed significant enhancement in emission intensity.

## 4. Conclusions

Herein, we report the synthetic procedures and the spectroscopic and single crystal X-ray diffraction characteristics for a series of lanthanide complexes containing dipyrido[3,2-*a*:2′,3′-*c*]phenazine (dppz) as ligand. Our synthetic approach is novel and is able to afford single crystals suitable for X-ray diffraction analysis for most of the compounds in the lanthanide series. X-ray analysis of the complexes reveals that all complexes are isomorphic, with the lanthanide ion coordinated by two dppz ligands and three nitrate anions. As it could be expected based on the T_1_ state of the ligand (18400 cm^−1^) and the resonant energy level of the studied lanthanides (Nd^3+^, Er^3+^, Yb^3+^), it was shown that the dppz ligand is an appropriate choice for the design of NIR emitting complexes. We have additionally studied the stability of the [Nd(NO_3_)_3_(dppz)_2_], [Er(NO_3_)_3_(dppz)_2_] and [Yb(NO_3_)_3_(dppz)_2_] complexes in solution over a period of time. No visible changes in the UV/Vis spectra were observed up to 6 h. Last, we have confirmed that the exemplary [Nd(NO_3_)_3_(dppz)_2_] complex shows DNA intercalation ability through an ethidium bromide displacement assay carried out with CT-DNA. These results point out that such carefully developed lanthanide polypyridyl complexes could serve as potential NIR luminescent probes for bioimaging applications and diagnostics.

## Figures and Tables

**Table 1:** Assignment of the labeled transitions a-d in the steady-state excitation and emission spectra of the Nd^3+^, Er^3+^ and Yb^3+^ complexes.

Excitation					Emission				
	Wavelength (nm)	Energy (cm^−1^)	Transition			Wavelength (nm)	Energy (cm^−1^)	Transition	
**NdL**									
a	465.5	21482	^2^D_3/2_, ^2^P_3/2_, ^2^G_9/2_, ^2^K_15/2_	←^4^I_9/2_	a	870.8	11484	^4^F_3/2_ →	^4^I_9/2_
b	512.2	19524	^4^G_9/2_, ^4^G_7/2_^2^K_13/2_	← ^4^I_11/2_	b	1052.7	9499		^4^I_11/2_
c	524.3	19073	^4^G_9/2_, ^4^G_7/2_, ^2^K_13/2_	←^4^I_9/2_	c	1334.5	7493		^4^I_13/2_
d	582.4	17170	^2^G_7/2_, ^4^G_5/2_	←^4^I_9/2_					
**ErL**									
no f-f transitions					a	1529.0	6548	^4^I_13/2_ →	^4^I_15/2_
**YbL**									
no f-f transitions					a	977.6	10229	^2^F_5/2_ →	^2^F_7/2_

## Supplementary Materials

The following are available online, Scheme S1: Synthesis of 1,10-phenanthroline-5,6-dione. Scheme S2: Synthesis of dipyrido[3,2-*a*:2′,3′-*c*]phenazine. Figure S1: Steady-state excitation spectrum of Eu(dppz)_2_(NO_3_)_3_, recorded at room temperature. Figure S2: Steady-state emission spectrum of Eu(dppz)_2_(NO_3_)_3_ at room temperature. Figure S3: UV-Vis absorption spectra of the [Er(dppz)_2_ (NO_3_)_3_] complex carried out over a range of 6 h in DMF. Figure S4: UV-Vis absorption spectra of the [Yb(dppz)_2_ (NO_3_)_3_] complex carried out over a range of 6 h in DMF. Figure S5: UV/Vis absorption spectrum of [Yb(NO_3_)_3_(dppz)_2_] complex dissolved in DMF: Tris buffer (5 mM Tris-HCl, 5 mM NaCl, pH = 7.2) in a 1: 1 ratio. The UV-Vis spectrum was recorded 2 h after dissolving the sample. Figure S6: UV-Vis absorption spectrum of [Yb(dppz)_2_(NO_3_)_3_] complex dissolved in Tris buffer (5 mM Tris-HCl, 5 mM NaCl, pH = 7.2). The UV/Vis spectrum was recorded 2 h after dissolving the sample.
