# Supplementary material for: DNA Intercalating Near-Infrared Luminescent Lanthanide Complexes Containing Dipyrido[3,2-a:2′,3′-c]phenazine (dppz) Ligands: Synthesis, Crystal Structures, Stability, Luminescence Properties and CT-DNA Interaction
Source: Molecules. 2020 Nov 13;25(22):5309. doi: 10.3390/molecules25225309 (PMC7697401; doi:10.3390/molecules25225309)
Supplement: Supplementary file 1 [file molecules-25-05309-s001.pdf]

Supporting Information

DNA intercalating near-infrared luminescent lanthanide complexes containing dipyrido[3,2-a:2′,3′-c]phenazine (dppz) ligands: synthesis, crystal structures, stability, luminescence properties and CT-DNA interaction

Aleksandar Savić[1,3], Anna M. Kaczmarek[1,4], Rik Van Deun[1,*], Kristof Van Hecke[2,*]

Synthesis of the ligand precursor 1,10-phenanthroline-5,6-dione (phendione)

1,10-Phenantroline-5,6-dione was obtained by oxidation of 1,10-phenanthroline with potassium bromate and 65% sulphuric acid at room temperature (Scheme S1).

1,10-Phenanthroline (55.5 mmol) was added in small portions under stirring to sulfuric acid (65%, 80 mL) and left to dissolve at room temperature. Potassium bromate (62 mmol) was added in portions over a period of 3 hours and the mixture was stirred at room temperature for 24 hours. Then, the mixture was poured over ice and was neutralized to pH 7 using sodium carbonate. The mixture was extracted with chloroform and the organic phase was dried over magnesium sulfate during the night. The solution was then filtered and evaporated to dryness. The crude product was recrystallized from methanol to obtain the desired yellow product.

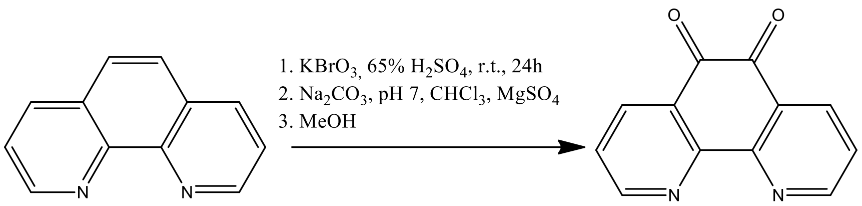

Scheme S1. Synthesis of 1,10-phenanthroline-5,6-dione.

Synthesis of the ligand, dipyrido[3,2-a:2′,3′-c]phenazine (dppz)

The dipyrido[3,2-a:2′,3′-c]phenazine (dppz) ligand was synthesized by a reaction between 1,10-phenanthroline-5,6-dione and *o*-phenylenediamine in 96% ethanol (Scheme S2).

Phendione (0.24 mmol) was dissolved in ethanol (96%, 4 mL) at 75 °C, *o*-phenylenediamine (0.24 mmol) was added and the mixture was heated for 30 minutes under reflux. After cooling to room temperature, the dppz ligand was precipitated. The product was isolated, washed with methanol and dried under vacuum.

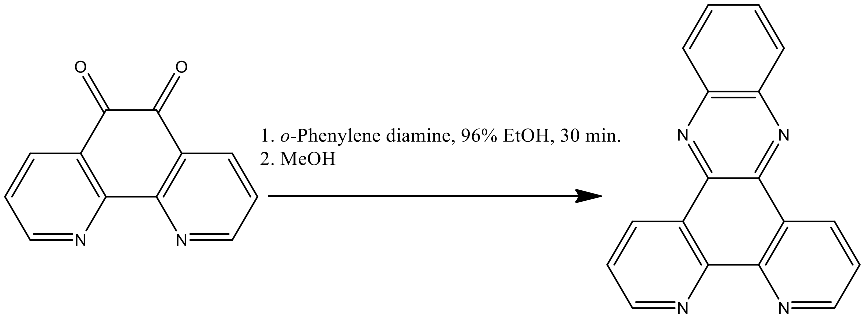

Scheme S2. Synthesis of dipyrido[3,2-a:2′,3′-c]phenazine.

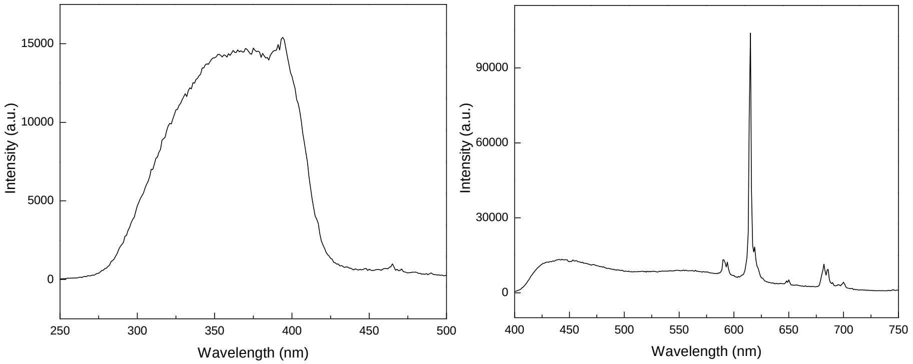

Figure S1 Steady-state excitation (left) and emission (right) spectra of [Eu(NO_3)$_3$(dppz)$_2$], recorded at room temperature.

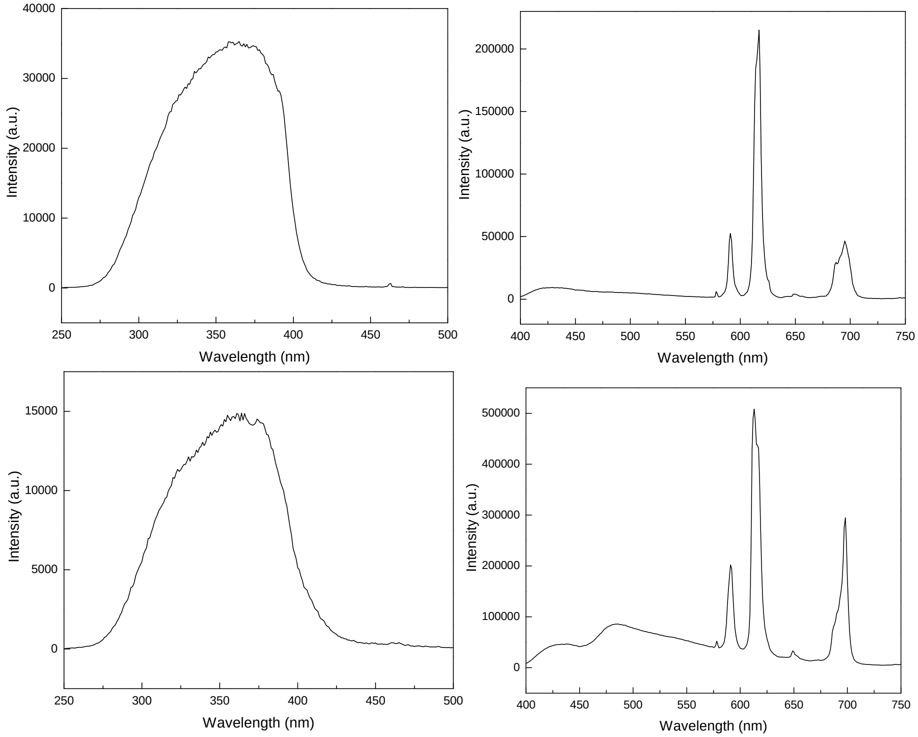

Figure S2 Excitation and emission spectra of [Eu(NO_3)$_3$(dppz)$_2$], recorded at room temperature in DMF (top spectra) and 50%DMF+50%H_2O (bottom spectra).

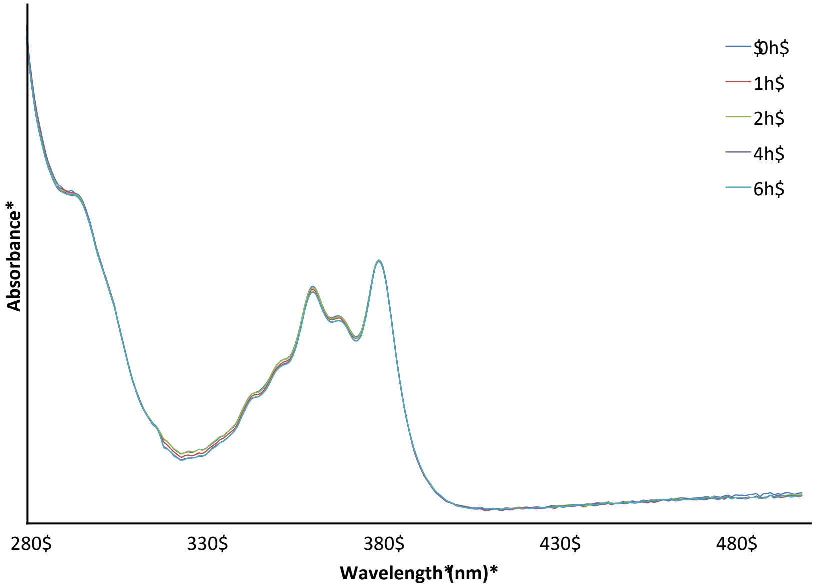

Figure S3 UV-Vis absorption spectra of the $[Er(NO_3)_3(dppz)_2]$ complex carried out over a range of 6 h in DMF.

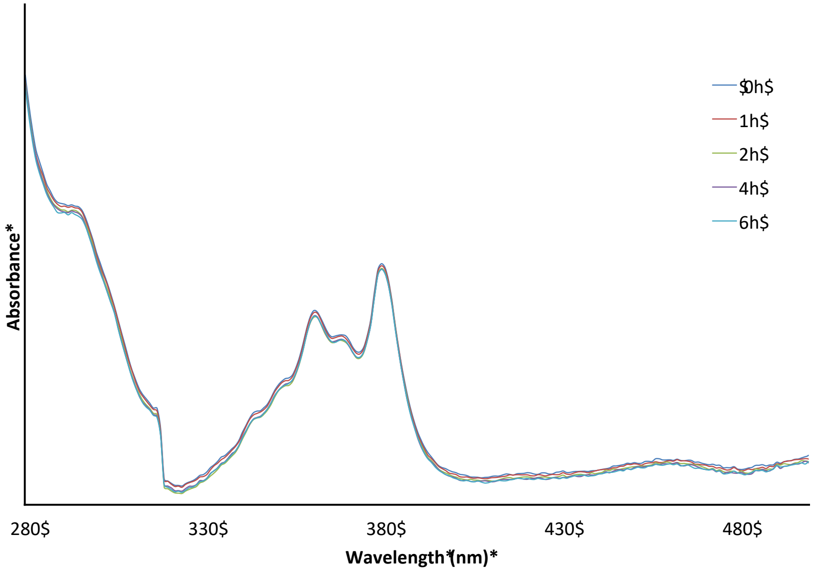

Figure S4 UV-Vis absorption spectra of the $[Yb(NO_3)_3(dppz)_2]$ complex carried out over a range of 6 h in DMF.

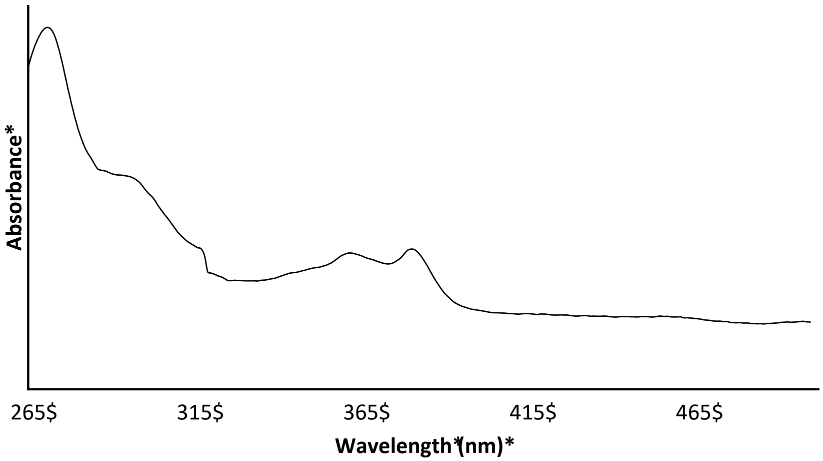

Figure S5 UV-Vis absorption spectrum of $[Yb(NO_3)_3(dppz)_2]$ complex dissolved in DMF : Tris buffer (5 mM Tris-HCl, 5 mM NaCl , pH = 7.2) in a 1 : 1 ratio. The UV-Vis spectrum was recorded 2 h after dissolving the sample.

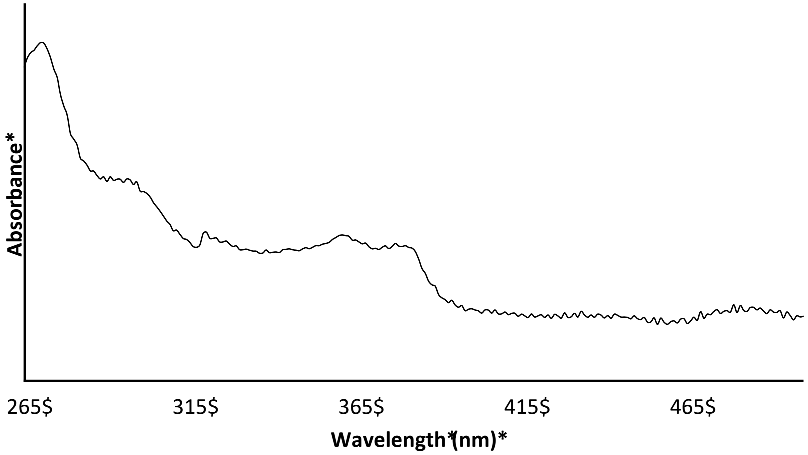

Figure S6 UV-Vis absorption spectrum of $[Yb(NO_3)_3(dppz)_2]$ complex dissolved in Tris buffer (5 mM Tris-HCl, 5 mM NaCl, pH = 7.2). The UV-Vis spectrum was recorded 2 h after dissolving the sample.

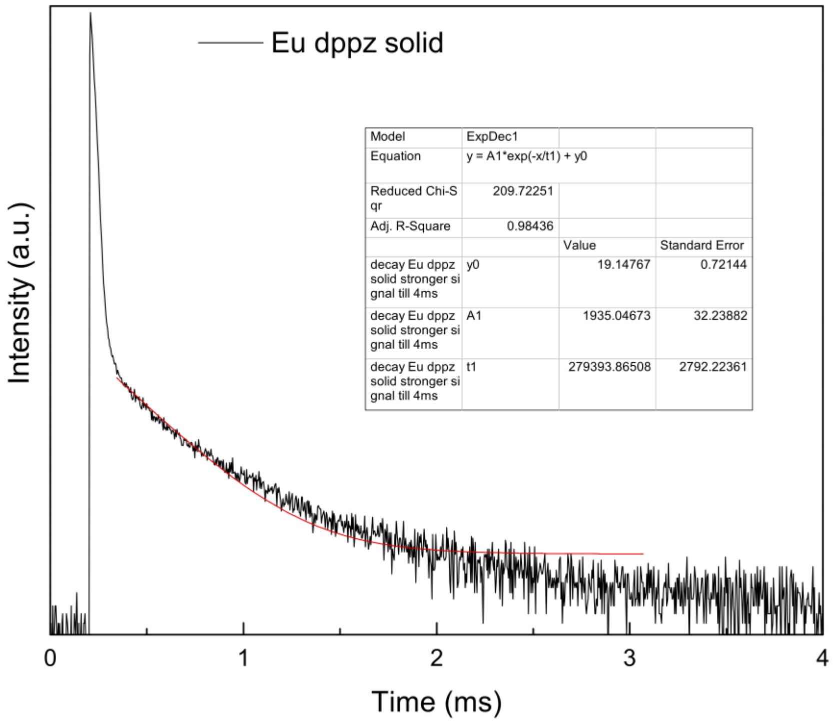

Figure S7 Decay profile of the $[Eu(NO_3)_3(dppz)_2]$ complex in the solid state.

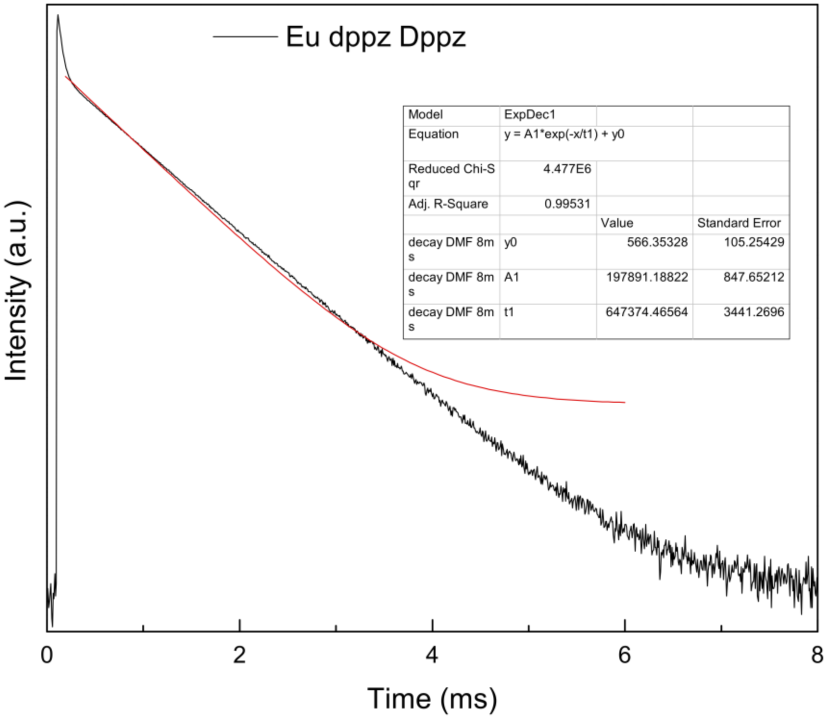

Figure S8 Decay profile of the $[Eu(NO_3)_3(dppz)_2]$ complex in DMF.

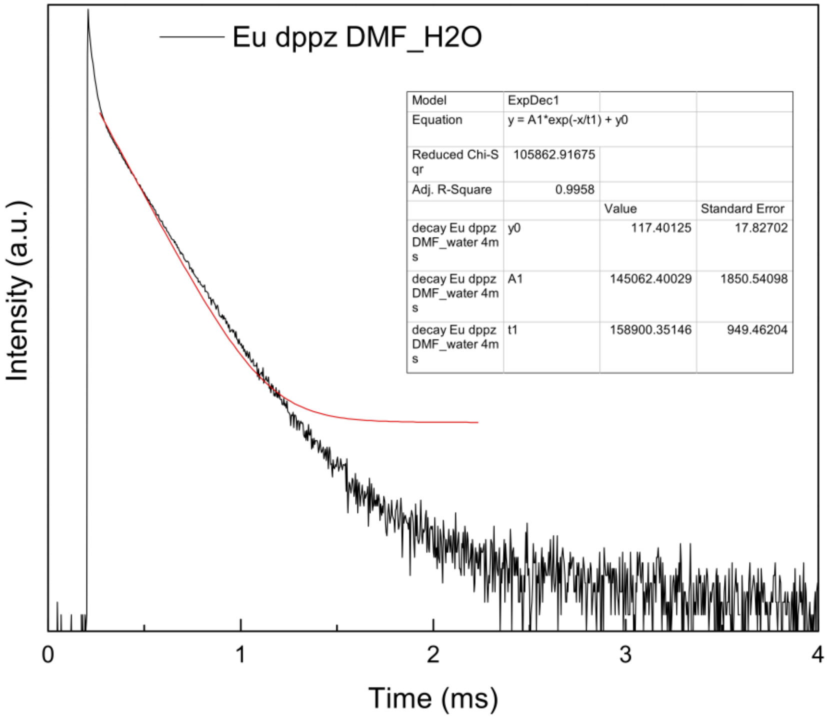

Figure S9 Decay profile of the $[Eu(NO_3)_3(dppz)_2]$ complex in DMF/H_2O 50/50.

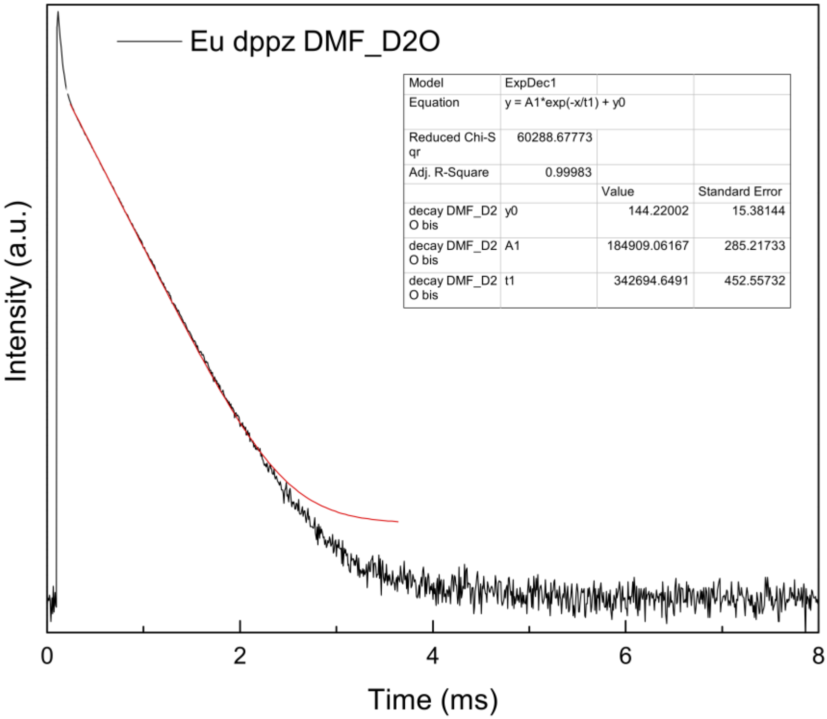

Figure S10 Decay profile of the $[Eu(NO_3)_3(dppz)_2]$ complex in DMF/D_2O 50/50.

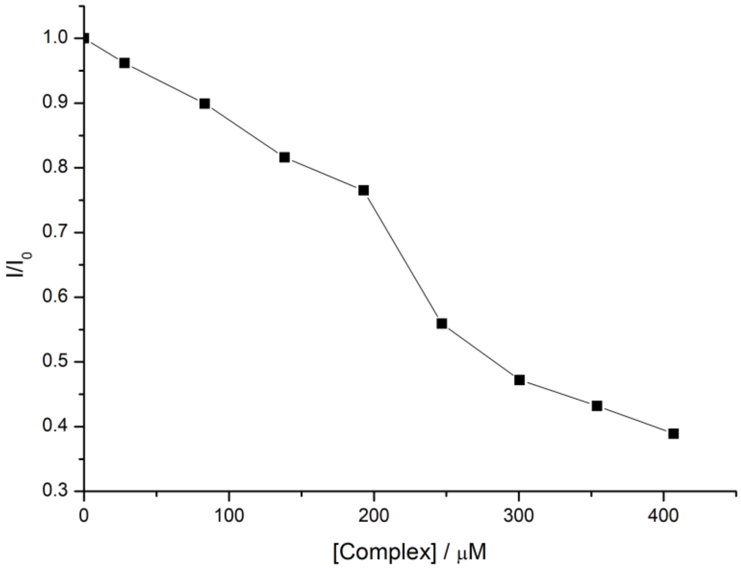

Figure S11 Stern-Volmer plot of I/I_0 *vs* [complex] for the [Nd(NO_3)$_3$(dppz)$_2$] complex for the EthB displacement assay with CT-DNA.
